# Solvation free energy in governing equations for DNA hybridization, protein–ligand binding, and protein folding

**DOI:** 10.1002/2211-5463.13897

**Published:** 2024-09-17

**Authors:** Caroline Harmon, Austin Bui, Jasmin M. Espejo, Marc Gancayco, Jennifer M. Le, Juan Rangel, Daryl K. Eggers

**Affiliations:** ^1^ Chemistry Department San José State University San José CA USA

**Keywords:** bulk water, calorimetry, desolvation, DNA duplex, hydration, ribonuclease inhibitor

## Abstract

This work examines the thermodynamics of model biomolecular interactions using a governing equation that accounts for the participation of bulk water in the equilibria. In the first example, the binding affinities of two DNA duplexes, one of nine and one of 10 base pairs in length, are measured and characterized by isothermal titration calorimetry (ITC) as a function of concentration. The results indicate that the change in solvation free energy that accompanies duplex formation (Δ*G* ^S^) is large and unfavorable. The duplex with the larger number of G:C pairings yields the largest change in solvation free energy, Δ*G* ^S^ = +460 kcal·mol^−1^per base pair at 25 °C. A van't Hoff analysis of the data is complicated by the varying degree of intramolecular base stacking within each DNA strand as a function of temperature. A modeling study reveals how the solvation free energy alters the output of a typical ITC experiment and leads to a good, though misleading, fit to the classical equilibrium equation. The same thermodynamic framework is applied to a model protein–ligand interaction, the binding of ribonuclease A with the nucleotide inhibitor 3′‐UMP, and to a conformational equilibrium, the change in tertiary structure of α‐lactalbumin in molar guanidinium chloride solutions. The ribonuclease study yields a value of Δ*G* ^S^ = +160 kcal·mol^−1^, whereas the folding equilibrium yields Δ*G* ^S^ ≈ 0, an apparent characteristic of hydrophobic interactions. These examples provide insight on the role of solvation energy in binding equilibria and suggest a pivot in the fundamental application of thermodynamics to solution chemistry.

## Graphical abstract

A new thermodynamic framework is examined that accounts for the release of solvation waters when two biological surfaces make contact. The change in solvation free energy was found to be relatively large and unfavorable for DNA hybridization and for the model protein–ligand interactions examined, whereas the change in solvation free energy was negligible for a tested protein conformational equilibrium.

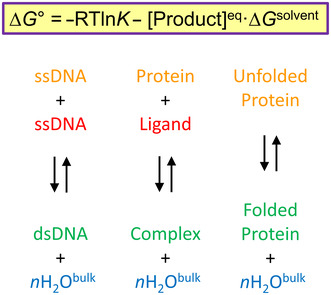

**Abbreviations**
CD: circular dichroism spectroscopy
GuHCl: guanidinium chloride
ITC: isothermal titration calorimetry
RNase A: ribonuclease A
ssDNA: single‐stranded DNA oligonucleotide

The stable binding of two molecules to form an active complex is a fundamental step in many biochemical reactions. Unfortunately, the classical thermodynamic equations for binding equilibria may not account fully for the contribution of the solvent. Because a complex between two molecules will interact with fewer water molecules than the unbound reactants, and because the chemical potential of a water molecule depends on the chemistry of any neighboring reactants or secondary solutes, changes in the free energy of water are expected to play a significant role in the thermodynamics of binding and conformational equilibria.

In 2013, Castellano and Eggers introduced a modified equation for binding equilibria that was derived from the chemical potential of all reaction participants, including the solvent [1]. The thermodynamic framework, elaborated further in a subsequent paper [2], captures the change in free energy associated with the subset of water molecules that are released from the surfaces of the reactants upon binding. The framework has been applied to multiple host–guest pairings [2, 3], in addition to evaluating the effects of secondary solutes on binding equilibria [4].

The thermodynamic framework is summarized by Eqns (1–5) below. For a binding reaction between two molecules, A and B, the following relationship is obtained for the overall change in reaction free energy when water is considered explicitly:

(1) $$\Delta G^{\mathrm{rxn}}=\mathrm{RT}\ln\frac{\left[\mathrm{AB}\right]}{\left[A\right]\left[B\right]}+\left[\mathrm{AB}\right]\Delta G^{S}+\Delta G{}^{\circ}$$

where *R* is the gas constant, *T* is the temperature in Kelvin, [AB] is the concentration of binary complex, Δ*G* ^S^ is the change in solvation free energy, and Δ*G*° is the standard‐state free energy constant. In general, the value of Δ*G* ^S^ reflects all linked reaction equilibria that are not represented by the concentration ratio of products to reactants [4]. For the current study, Δ*G* ^S^ is expected to be dominated by the solvent‐associated term, denoted $\Delta G^{H_{2}O}$ in some of our previous work, and defined by the following:

(2) $$\Delta G^{S}=\Delta G^{\text{solvent}}=n\left({\bar{G}}^{\text{bulk}}-{\bar{G}}^{\text{surface}}\right)$$

where “*n*” is the number of water molecules released per mole of complex formed, and where the bar above ${\bar{G}}^{\text{bulk}}$ and ${\bar{G}}^{\text{surface}}$ is a reminder that these represent location‐averaged free energy values for a single water molecule in the bulk phase or next to a reactant surface, respectively. Because “*n*” is defined as the number of water molecules released per mole of formed complex, the total contribution of water to the reaction free energy change is given by the term [AB]·Δ*G* ^S^, as found in Eqn (1). For the experiments that follow, the number of water molecules that contribute to Δ*G* ^S^ is unknown; this thermodynamic treatment estimates the total change in solvation energy upon binding with no assumptions regarding the detailed solvation state of each reactant. Of special note, the presence of ${\bar{G}}^{\text{bulk}}$ in the definition of Δ*G* ^S^ (Eqn 2) enables one to account for changes in equilibria due to the addition of secondary solutes that alter ${\bar{G}}^{\text{bulk}}$ through cosolute–water interactions [4].

The standard‐state free energy in Eqn (1), Δ*G*°, is obtained from combining all of the constant terms in the derivation that arise from the traditional reactants, leading to the following:

(3) $$\Delta G^{\circ }=\left(\mu^{\circ ,AB}-\mu^{\circ ,A}-\mu^{\circ ,B}\right)+\mathrm{RT}\ln\frac{\gamma_{i}^{\mathrm{AB}}}{\gamma_{i}^{A}\gamma_{i}^{B}}-\mathrm{RT}\ln\frac{\left[\mathrm{AB}\right]{}^{\circ}}{\left[A\right]{}^{\circ}\left[B\right]{}^{\circ}}$$

where *μ*°^,*x*^ are the standard‐state potentials, $\gamma_{i}^{x}$ are the activity coefficients in solution *i*, and [*x*]° are the reference concentrations of each species *x*. Note that $\gamma_{i}^{x}$ and [*x*]° are the constant terms for each reactant from the definition of thermodynamic activity, $a_{i}^{x}$:

(4) $$a_{i}^{x}=\gamma_{i}^{x}\left(\frac{{\left[x\right]}_{i}}{\left[x\right]{}^{\circ}}\right)$$

At equilibrium, Δ*G* ^rxn^ = 0, and Eqn (1) may be reduced to the governing relationship:

(5) $$\Delta G{}^{\circ}=-\mathrm{RT}\ln K-{\left[\mathrm{AB}\right]}^{\mathrm{eq}}\Delta G^{S}$$

With regard to Eqn (5), the equilibrium quotient, *K*, is defined as the ratio of complex to free reactants at equilibrium in the direction of association. As with the classical approach, all concentrations are treated as dimensionless quantities due to division by a reference concentration in the derivation, but the concentrations must be inserted in the same units, typically molarity for dilute solutions. Because Δ*G*° and Δ*G* ^S^ are constants in Eqn (5), this relationship predicts that the equilibrium quotient will change with increasing product formation when the solvation free energy is of significant magnitude; *K* is not a constant unless the change in solvation free energy is zero.

The derivation of Eqn (5) has been questioned by those who contend that the chemical potential of all water molecules in a solution at equilibrium must be equal, and, therefore, Δ*G* ^S^ must be zero. This notion, however, may be misleading. The change in free energy of the system at equilibrium (reactants plus solvent) is zero, but this stipulation does not preclude the existence of subpopulations of water molecules that differ in chemical potential due to neighboring solutes (i.e., boundary conditions). With respect to the solvent, a state of equilibrium means only that the movement of a water molecule from one position to a location of differing chemical potential must be countered by a second water molecule that moves in the opposite direction. Water molecules should be viewed as existing in a dynamic equilibrium between the hydration spheres of the reacting species and all other locations that comprise the bulk phase [4]. The time‐averaged potential of all water molecules is the same, but the location‐specific potential at a given instant in time will depend on the presence or absence of neighboring solutes.

Another common misconception is that all concentration‐dependent changes in equilibria, as predicted by Eqn (5), should be attributed to changes in the activity coefficients of the reactants. This explanation presumes—often without evidence—that self‐interacting species (i.e., reactant A with reactant A) are responsible for the observed changes in equilibria. However, this rationale does not seem plausible when the reactant concentrations are in the millimolar range or below. For a small reacting species at a concentration of 10 mm, the ratio of water molecules to reactant molecules is approximately 5500 : 1, and water–reactant interactions should far outnumber reactant–reactant interactions [3, 4]. In the current work, the ratio of excess water to reactant will be reduced due to the excluded volume of the macromolecules, but the employed concentrations of macromolecule are also reduced, rarely exceeding 1.0 mm. Thus, reactant–reactant interactions remain a rare event compared to reactant–solvent interactions in the present study, and the activity coefficient of each reactant is expected to be a constant within the tested concentration range.

The results that follow attribute concentration‐dependent changes in binding equilibria to the concomitant changes in solvation, as governed by Eqn (5). This relationship can be tested by measuring the equilibrium quotient as a function of product concentration. If the experimental data agree with the governing equation, then a plot of −*RT*ln*K* versus [AB], the concentration of complex at equilibrium, should yield a straight line with a slope equal to Δ*G* ^S^ and a *y*‐intercept equal to Δ*G*°. For the first time, this thermodynamic framework is applied here to model biomolecular interactions that involve DNA oligonucleotides and proteins.

## Materials and methods

### Reagents

All single‐stranded DNA oligonucleotides (ssDNA) were purchased from Integrated DNA Technologies, Coralville, Iowa, USA. The concentrations of stock ssDNA solutions were checked prior to each ITC run using a Cary 60 spectrophotometer. The DNA buffer consisted of 100 mm NaCl, 0.10 mm EDTA, and 10 mm sodium phosphate, pH 6.8. The oligonucleotide names, corresponding sequences, and extinction coefficients are listed below:

oligo9: 5′ – CAA ATA AAG – 3′; ε_260_ = 100 900 m ^−1^·cm^−1^.

oligo9c: 5′ – CTT TAT TTG – 3′; ε_260_ = 80 000 m ^−1^·cm^−1^.

oligo10: 5′ – ATG CTG ATG C – 3′; ε_260_ = 94 800 m ^−1^·cm^−1^.

oligo10c: 5′ – GCA TCA GCA T – 3′; ε_260_ = 97 000 m ^−1^·cm^−1^.

Ribonuclease A from bovine pancreas (Fisher Scientific, Waltham, MA, USA, #BP2539) and α‐lactalbumin from bovine milk (Sigma‐Aldrich, St. Louis, MO, USA, #L6010) were used for protein studies. Protein concentrations were measured by spectrophotometry using extinction coefficients of 9800 m ^−1^·cm^−1^ at 278 nm (RNase A) and 28 500 m ^−1^·cm^−1^ at 282 nm (α‐lactalbumin). The ribonuclease inhibitor, uridine 3′‐monophosphate (3′‐UMP), was purchased as a disodium salt from Biosynth, Gardner, MA, USA (#NU07383), and concentrations were determined by using an extinction coefficient of 9780 m ^−1^·cm^−1^ at 262 nm, as reported for 5′‐UMP [5].

### Calorimetry

Isothermal titration calorimetry (ITC) was performed with a Microcal instrument, model VP‐ITC, using the analysis programs provided by the manufacturer. All solutions were degassed briefly under vacuum (ThermoVac by MicroCal for Malvern Panalytical, Westborough, MA, USA). Prior to each calorimetry run, the sample cell (~ 1.45 mL volume) was cleaned and rinsed with one volume of the corresponding sample buffer. For DNA trials, the injection syringe (~ 0.28 mL) was filled with the complementary oligonucleotide at a concentration 20‐fold higher than the cell for oligo9 experiments and 10‐fold higher for oligo10 experiments. For RNase A trials, stock protein solutions were made fresh and 3′‐UMP stocks were stored at −20 °C, both in a buffer solution of 25 mm Bis‐Tris with 25 mm KCl adjusted to pH 6.0. The sample cell was loaded with the desired RNase A concentration, and the injection syringe contained 3′‐UMP at a concentration 10‐ or 20‐fold higher than RNase A. The typical ITC protocol consists of 28 injections of 10 μL volume or 55 injections of 5 μL volume. In all trials, the first injection was set at 2 μL, and the first titration peak was discarded from the analysis due to the unavoidable error in the injection volume after filling and moving the syringe to the calorimeter base. A background ITC file, or a constant corresponding to the background injection enthalpy, was subtracted from each integrated peak prior to analysis.

### Circular dichroism spectroscopy

A 100 mg·mL^−1^ stock solution of α‐lactalbumin was prepared in 10 mm Tris buffer (pH 7.0) containing 10 mm EDTA and 1.25 or 3.0 m guanidine HCl (Fisher). The stock protein solution was diluted to the desired concentrations in the same buffer and transferred to cuvettes of varying pathlengths such that the product of c·*l* from Beer's law was constant. Wavelength scans were carried out on an Aviv Model 215 circular dichroism spectrometer at 25 °C in the near‐UV region (250–320 nm). Five scans per sample were taken and averaged. A control buffer solution was subtracted from each result before exporting the data to an Excel spreadsheet for analysis.

### Modeling the governing equation

Equation 5 was modeled by setting the total concentrations of the ssDNA (*X*_t_) and its complement (*M*_t_) as known inputs after each titration point and by using a mass balance expression to replace the concentration of the free ssDNA in the governing equation, where *Z* is the unknown concentration of duplex at equilibrium:

$$\left[\text{ssDNA}\right]=X_{t}-Z$$

$$\left[\text{ssDNAc}\right]=M_{t}-Z$$

The value of *X*_t_ after each injection was adjusted by a constant increment, set up as an array of 28 injections of 10 μL volume at a concentration 10‐fold higher than the starting concentration of ssDNA oligonucleotide in the cell, *M*_t_. The two free energy parameters, Δ*G*° and Δ*G* ^S^, were set as constants that approximate the experimentally‐obtained values for oligo10/10c binding. The concentration of bound complex (*Z*) was solved by WolframAlpha after each injection from the following expression, in accord with Eqn (5):

(6) $$\mathrm{\Delta G}{}^{\circ}=-\mathrm{RT}\cdot \ln\left(\frac{Z}{\left(X_{t}-Z\right)\left(M_{t}-Z\right)}\right)-Z\cdot \Delta G^{S}$$

Typically, two or three roots are obtained from WolframAlpha, but only one root is a real number below the maximum possible concentration. After solving for *Z*, the equilibrium quotient within the natural logarithm term is calculated, as well as the heat released after each injection. The heat may be estimated by multiplying a constant molar binding enthalpy by the increase in *Z* relative to the previous injection. See data accessibility statement for link to further modeling details.

## Results

In order to estimate the value of Δ*G* ^S^ from experimental data, the last term in the Eqn (5), [AB]·Δ*G* ^S^, must be of sufficient magnitude to observe a shift in the equilibrium. For DNA and proteins, this presents a challenge because, depending on the value of Δ*G* ^S^, it may not be possible to push the concentration of the macromolecule high enough to observe an effect. In previous work employing small molecules, the ideal upper concentration varied from 3 to 10 mm [3]. With this issue in mind, the following studies were carried out to assess the feasibility of detecting a concentration‐dependent change in each equilibrium, as predicted by the governing equation.

### Framework applied to DNA hybridization

**Fig. 1:**
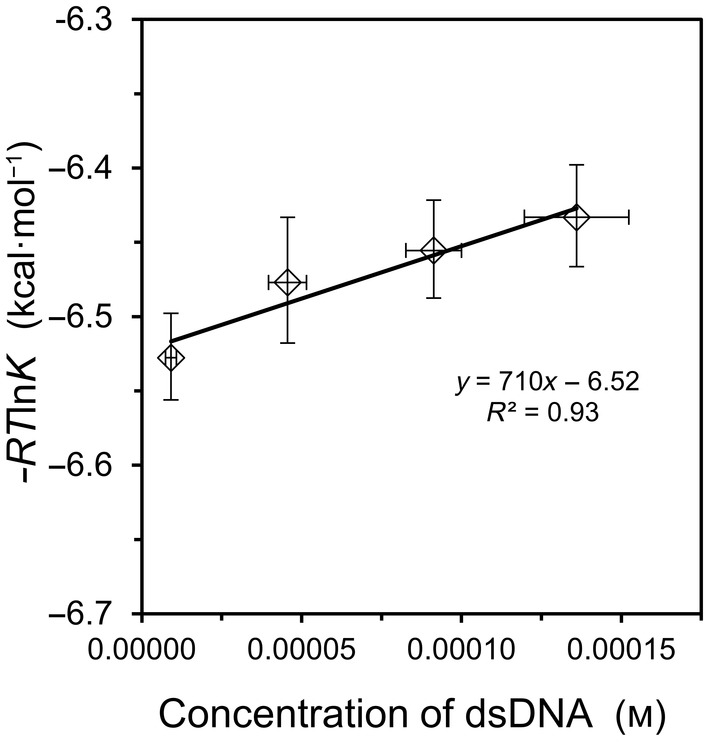
Hybridization of oligo9 with oligo9c at 25 °C. Equilibrium binding quotients were obtained by ITC as a function of concentration. The linear fit is indicated on the graph, from which the slope corresponds to Δ*G* ^S^ and the *y*‐intercept corresponds to Δ*G*°, in accord with Eqn (7). Error bars represent standard deviation values (*n* = 3). See Table S1 for corresponding values of *K* and Δ*H* ^ITC^.

In the case of DNA hybridization, Eqn (5) may be expressed as follows:

(7) $$\Delta G{}^{\circ}=-\mathrm{RT}\ln\left(\frac{\left[\text{dsDNA}\right]}{\left[\text{ssDNA}\right]\left[\text{ssDNAc}\right]}\right)-\left[\text{dsDNA}\right]\Delta G^{S}$$

where [dsDNA] is the concentration of double‐stranded product at equilibrium, and where [ssDNA] and [ssDNAc] represent the equilibrium concentrations of the single‐stranded DNA and its complement, respectively. Because ITC is best reserved for binding affinities in the range of *K* = 10^4^ to 10^7^, short DNA oligonucleotides were employed for the hybridization studies. Figure 1 summarizes the result for the shorter of two model reactions, oligo9 with oligo9c. The measured value of *K* declined modestly from 6.1 × 10^4^ at 0.010 mm duplex to 5.2 × 10^4^ at 0.150 mm duplex. This same duplex has been studied previously, but the prior investigations used a high salt concentration of 1.0 m NaCl which precludes a meaningful comparison of *K* values to the current work undertaken at a 10‐fold lower salt concentration [6, 7]. As seen in Fig. 1, the data for oligo9/9c binding yield a reasonable fit to Eqn (7), leading to free energy values of Δ*G*° = −6.52 ± 0.04 and Δ*G* ^S^ = +710 ± 500 kcal·mol^−1^. The large uncertainty in Δ*G* ^S^ is an unavoidable outcome of the low and narrow concentration range employed.

**Fig. 2:**
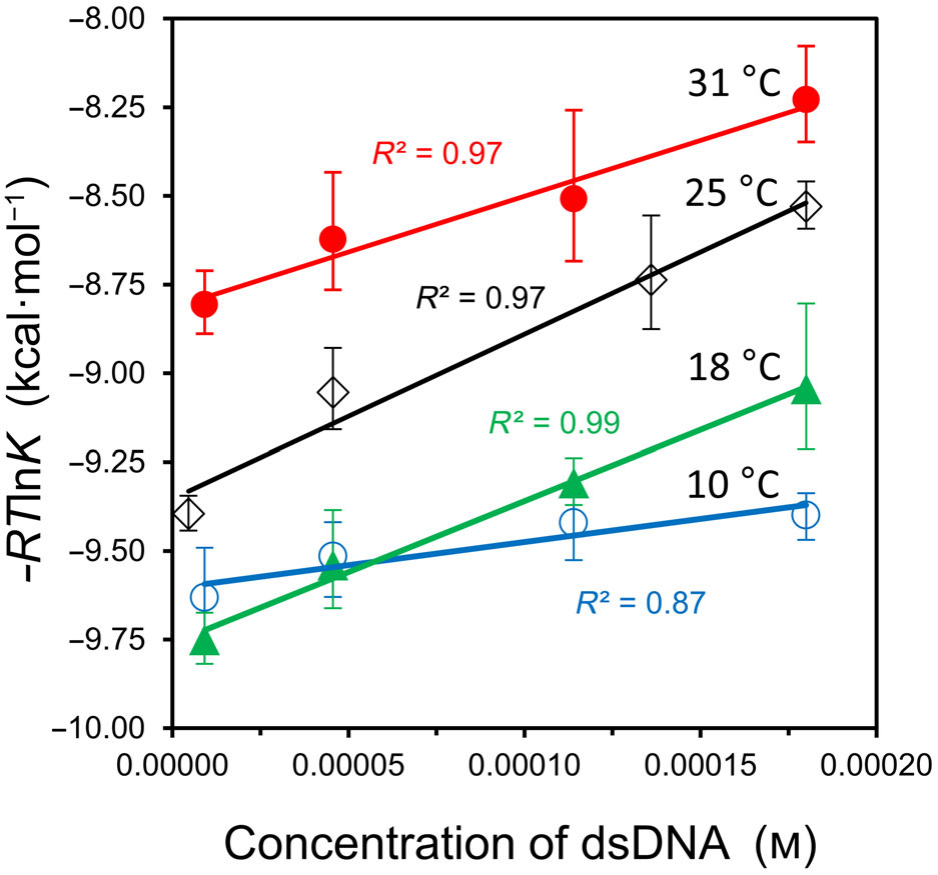
Hybridization of oligo10 with oligo10c. The corresponding temperatures and goodness of fits to Eqn (7) are shown on the graph. Note that the starting conformations of the oligonucleotides vary with temperature due to different amounts of intramolecular stacking. Error bars represent standard deviation values (*n* = 4). See Fig. S1 for sample ITC data and Table S2 for corresponding values of *K* and Δ*H* ^ITC^.

In a second test of hybridization, a DNA duplex of stronger binding affinity was employed. Oligo10 and oligo10c form five G:C base pairs, as opposed to just two for oligo9/9c, and a binding affinity on the order of 10^6^ has been reported for this duplex, as measured by ITC under similar conditions [8, 9]. In the current study, the concentration range was expanded to an upper value of 0.200 mm, and the ITC experiments were performed at four different temperatures. As shown in Fig. 2, a reasonable fit to Eqn (7) was observed at each temperature. For the specific dataset at 25 °C, the value of *K* decreased from 7.72 × 10^6^ at the lowest concentration to 1.79 × 10^6^ at the highest concentration, a much broader range than observed with oligo9/9c. These *K* values are consistent with a reported value of 3.5 × 10^6^, as obtained from the average of multiple measurements in the concentration range of 0.004–0.051 mm [9].

**Fig. 3:**
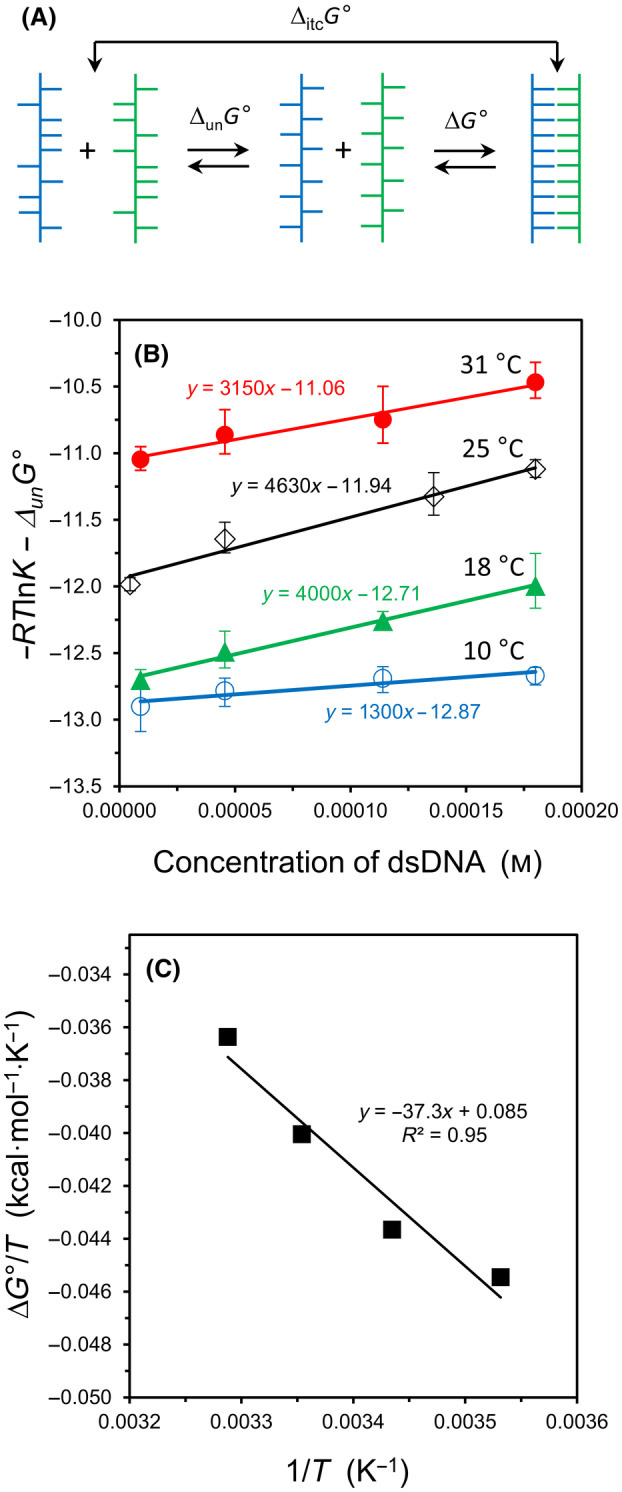
Revised analysis of oligo10/10c hybridization. (A) Schematic diagram of two‐step process for unstacking of intramolecular bases prior to duplex formation; Δ_itc_ *G*° = Δ_un_ *G*° + Δ*G*°. (B) Updated graph for hybridization of oligo10/10c using Δ*G*° values in Table 1. Error bars represent standard deviation values, *n* = 4. (C) A van't Hoff analysis of the Δ*G*° values in Table 1.

The intersection of datasets at 10 °C and 18 °C in Fig. 2 was initially viewed as curious and unexpected. After further reflection, however, it was realized that the datasets in Fig. 2 are influenced by intramolecular base stacking within each single‐stranded oligonucleotide which also varies with temperature (Fig. 3A). This residual structure has been detected and quantified by others with differential scanning calorimetry [9, 10, 11]. Thus, the starting point for hybridization is different at each temperature; a lower temperature begins with more intramolecular stacking than a higher temperature.

To place the temperature‐dependent results on the same starting basis, the free energy required to unstack each oligonucleotide (Δ_un_ *G°*) was calculated using the reported standard‐state values for the enthalpy and entropy of unstacking for oligo10 and oligo10c [9, 12]. The values of Δ*G*° obtained after subtracting the free energy of unstacking from the ITC‐measured value (Δ_itc_ *G°*) are summarized in Table 1.

**Table 1:** Accounting for the free energy of unstacking.^a^ ^,^ ^b^ ^,^ ^c^

*T* (°C)	Δ_un_ *G*° = Δ_un_ *H*°−*T*Δ_un_ *S*°			Fraction stacked (*f*)	Δ_itc_ *G*° from Fig. 2	Δ*G*° = Δ_itc_ *G*° − *f*Δ_un_ *G*°
	oligo10	oligo10c	Sum			
10	+1.62	+1.65	+3.27	1.0	−9.60	−12.87
18	+1.45	+1.50	+2.95	1.0	−9.76	−12.71
25	+1.30	+1.37	+2.67	0.97	−9.35	−11.94
31	+1.18	+1.26	+2.44	0.92	−8.82	−11.06

^a^ Free energy units in kcal·mol^−1^

^b^ Values of Δ_un_ *H*° and Δ_un_ *S*° from Lang and Schwarz [9]

^c^ Fraction in stacked conformation estimated from Zhou et al. [12].

Presumably, the temperature‐specific value of Δ*G* ^S^ is also influenced by the unstacking transition of ssDNA, but the concentration dependence of the unstacking transition is unknown for oligo10 and oligo10c and – due to an expected weak signal – would be difficult to measure by differential scanning calorimetry at the lower concentrations utilized in this study. For this reason, the datasets in Fig. 2 were re‐plotted in Fig. 3B to reflect the new *y*‐intercepts (Δ*G*°) without altering the slopes (Δ*G* ^S^). The datasets at 10 °C and 18 °C no longer intersect in the revised graph, and a van't Hoff analysis of the Δ*G*° values leads to a hybridization enthalpy of Δ*H*° = −37 kcal·mol^−1^ and entropy of Δ*S*° = −0.085 kcal·mol^−1^·K^−1^, as shown in Fig. 3C. These energies correspond to oligonucleotides that start in a fully‐unstacked conformation. The Δ*H*° value is smaller than the ITC‐measured enthalpy of −47 ± 5 kcal·mol^−1^, as obtained from the average of all trials at 25 °C.

### Modeling of titration experiments

**Fig. 4:**
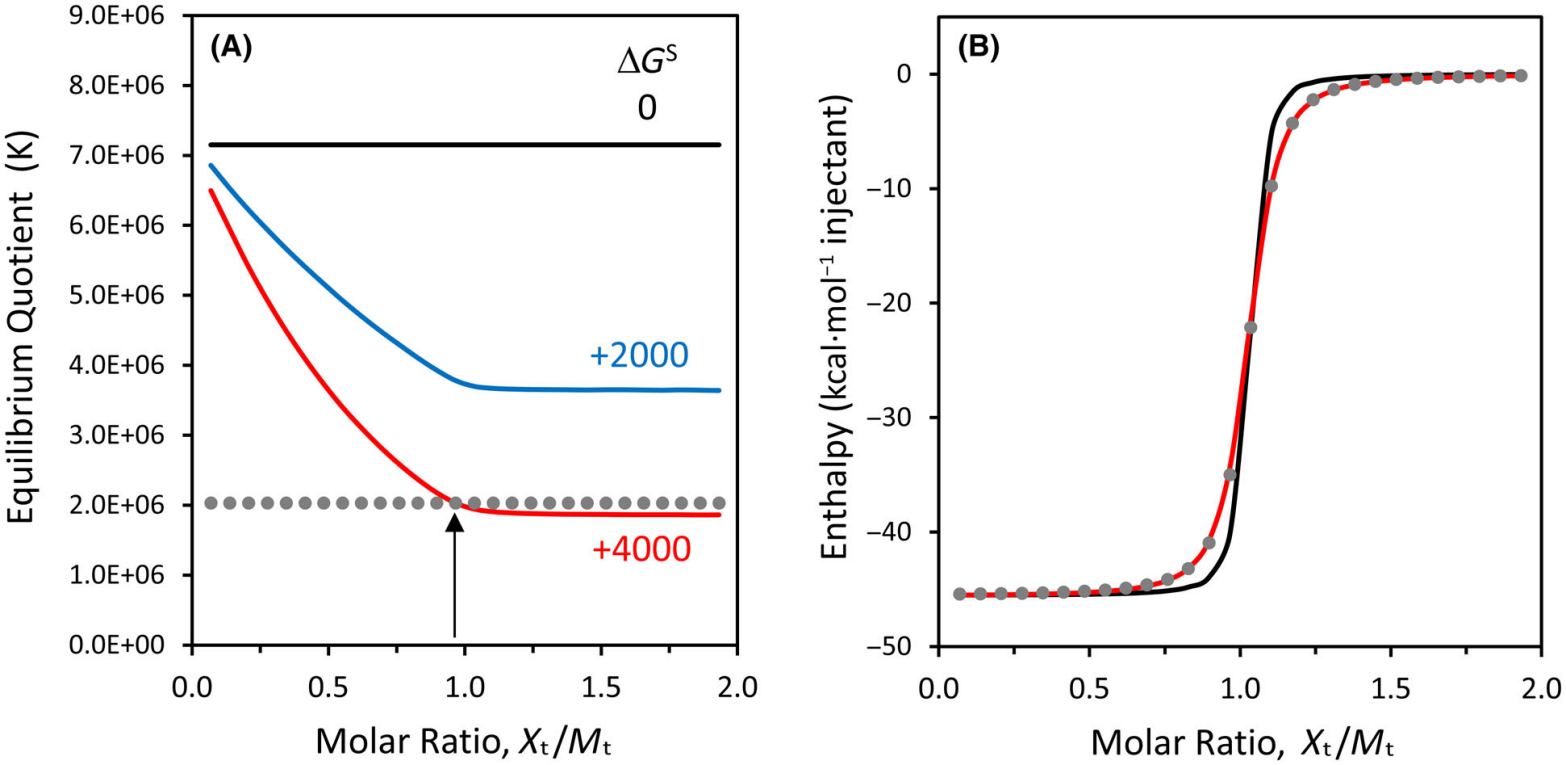
Modeling of titration results for oligo10/10c at 0.20 mm concentration and 25 °C. (A) Equilibrium quotients as a function of Δ*G*° and Δ*G* ^S^, as obtained from Eqn (6) using WolframAlpha to calculate the unknown concentration of duplex after each injection. The *x*‐axis represents the molar ratio of total injected species (*X*_t_) to total binding partner in the ITC cell (*M*_t_). For the three solid curves, Δ*G*° = −9.30 kcal·mol^−1^, and Δ*G* ^S^ is given next to each curve in kcal·mol^−1^. Simulations for Δ*G* ^S^ = 0 correspond to a constant value of *K*. The gray dotted line represents a constant *K* value of 2.03 × 10^6^, and the vertical arrow indicates the intersection with the dataset for Δ*G* ^S^ = +4000, just prior to exceeding a molar ratio of 1.0. (B) Simulated ITC enthalpy curves corresponding to datasets in panel (A). The gray dots represent *K* = 2.03 × 10^6^ (Δ*G* ^S^ = 0, Δ*G*° = −8.60 kcal·mol^−1^) and nearly coincide with the red curve (for which Δ*G* ^S^ = +4000, Δ*G*° = −9.30 kcal·mol^−1^).

One nuance of the thermodynamic framework employed here is that it predicts the equilibrium quotient should change after each injection during the progress of an isothermal titration experiment [3]. To demonstrate this phenomenon, a modeling study was performed with experimental parameters that approximate the hybridization of oligo10/10c at 0.20 mm concentration (Fig. 4). When the change in solvation free energy is positive, the equilibrium quotient declines after each injection for the first half of the titration, approaching a constant value near the midpoint of the titration, as shown in Fig. 4A. The midpoint corresponds to a 1 : 1 molar ratio in the total concentration of each oligonucleotide. The last half of the titration is characterized by a nearly‐constant value of *K* because the oligonucleotide that started in the calorimeter cell has become the limiting reactant and because nearly all of this limiting reactant has formed a duplex.

Simulated ITC enthalpy curves for Δ*G* ^S^ values of zero and +4000 kcal·mol^−1^ are shown in Fig. 4B. Note how the curve for Δ*G* ^S^ = +4000 (red solid line) coincides with a curve obtained from the classical equation when *K* is a constant (gray dots). This constant *K* value corresponds to the last injection prior to exceeding 1 : 1 molar ratio in the simulated curve for Δ*G* ^S^ = +4000 in Fig. 4A. For this reason, the *K* values obtained from the ITC instrument's fitting program, which utilizes the classical equation for binding equilibria, are matched with the concentration of the limiting reactant in the calorimeter cell near the 1 : 1 point, as explained in a previous study [3]. An experimental ITC run at the same concentration as the simulation may be found in Fig. S1D. Figure 4B is extraordinary for demonstrating how the classical binding equation may lead to a deceivingly good fit with ITC data when the equilibrium is changing markedly during the first half of the titration due to the effects of Δ*G* ^S^.

### Framework applied to protein–ligand interactions and protein folding

Ribonuclease A has served as a model protein for enzyme structure, enzyme function, and inhibitor binding studies for decades [13, 14]. A classical ITC study of protein–ligand interactions, carried out by J.F. Brandts and coworkers, examined the binding of RNase A with the nucleotide inhibitor 2′‐CMP and noted a significant decrease in the equilibrium ratio with increasing reactant concentration [15]. This rare example of a concentration‐dependent equilibrium was reported, in part, because the authors wanted to demonstrate the versatility of a new ITC instrument over a 50‐fold range in protein concentration. The authors hypothesized that the concentration‐dependent *K* values were due to dimerization or aggregation of RNase A, though a footnote in a subsequent report attributed the results to 2′‐CMP ‘if they arise from nonideality effects’ [16].

**Fig. 5:**
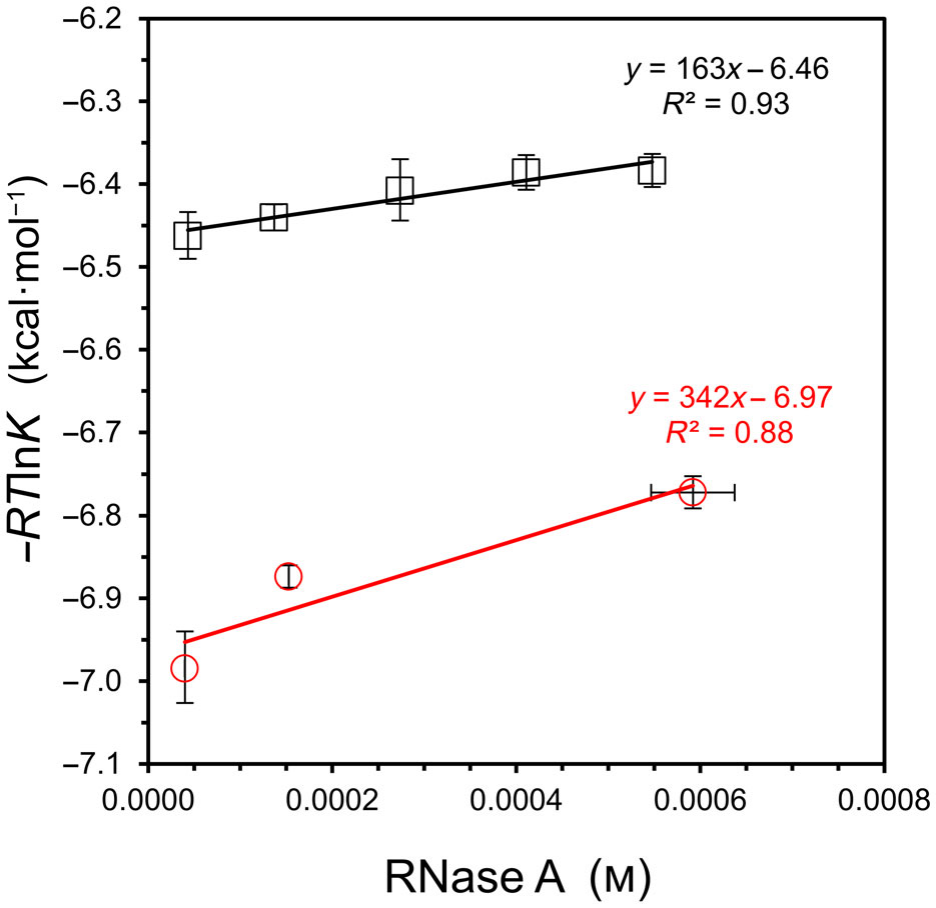
Binding analysis of 3′‐UMP/RNase A and 2′‐CMP/RNase A as a function of concentration. Binding of the 3′‐UMP inhibitor was measured in 25 mm KCl and 25 mm Bis‐Tris buffer, pH 6.0, at 25 °C (top, black squares). The *K* values for 2′‐CMP are tabulated in Wiseman et al., as measured in 200 mm KCl and 200 mm potassium acetate buffer, pH 5.5, at 28 °C (bottom, red circles) [15]. Error bars represent standard deviation values (*n* = 4). For 3′‐UMP, see Fig. S2 for sample ITC data and Table S3 for corresponding values of *K* and Δ*H* ^ITC^.

In another study employing RNase A, a salt‐dependent change in binding affinity with the inhibitor 3′‐UMP was observed, but the investigators increased the protein concentration simultaneously, making it impossible to separate solvation effects from salt effects [17]. In the current work, binding of 3′‐UMP to RNase A is re‐examined at five concentrations while maintaining a constant NaCl concentration (Fig. 5, upper line). For comparison, the 2′‐CMP binding result from the Brandts study is plotted on the same graph (Fig. 5, lower line). Because some of the experimental details are unknown for the Brandts dataset, it was assumed that the concentration of complex at the 1 : 1 molar titration point (*x*‐value on graph) is 90% of the starting concentration of RNase A in the calorimeter cell.

Although a concentration‐dependent trend is difficult to establish with high certainty in such a narrow range of protein concentration (highest concentration of 0.6 mm), a linear fit is observed in Fig. 5 for 3′‐UMP/RNase A, leading to binding free energies of Δ*G* ^S^ = +160 ± 100 kcal·mol^−1^ and Δ*G*° = −6.46 ± 0.03 kcal·mol^−1^. The estimated change in solvation energy for 2′‐CMP binding is about twice that of 3′‐UMP, but the number and distribution of tested concentrations are not ideal, leading to a weaker fit to the governing equation for the 2′‐CMP study (Fig. 5, bottom line).

In the last example, a conformational equilibrium is tested against the governing equation. Protein folding is often approximated as a 2‐state process, U↔F, where U is the unfolded or denatured state and F is the folded, native state of the protein. The two‐state assumption holds well for many smaller proteins, and reconfiguration of Eqn (5) to reflect a two‐state folding equilibrium leads to the following expression:

(8) $$\Delta G{}^{\circ}=-\mathrm{RT}\ln\frac{{\left[F\right]}^{\mathrm{eq}}}{{\left[U\right]}^{\mathrm{eq}}}-{\left[F\right]}^{\mathrm{eq}}\Delta G^{S}$$

One challenge in applying Eqn (8) to protein folding equilibria is the strong cooperativity of most folding transitions, leading to a narrow experimental window in which both species may be detected. In order to measure a concentration‐dependent change in the equilibrium of a model protein, the structure of α‐lactalbumin (123 amino acid residues) was examined in 1.25 m guanidinium chloride (GuHCl) by using circular dichroism (CD) in the near‐UV region to follow changes in tertiary structure. In this case, the unfolded state is a molten globule that retains some of its secondary structure, and the selected guanidinium concentration is near the midpoint of the two‐state transition for the Ca^2+^‐free apoprotein when following the CD spectrum at 270 nm [18]. To ensure that all protein is in the apo‐state, it was critical to include a higher‐than‐normal EDTA concentration of 10 mm in the stock solution (~ 7 mm protein) to chelate residual calcium ion from the freeze‐dried protein preparation.

**Fig. 6:**
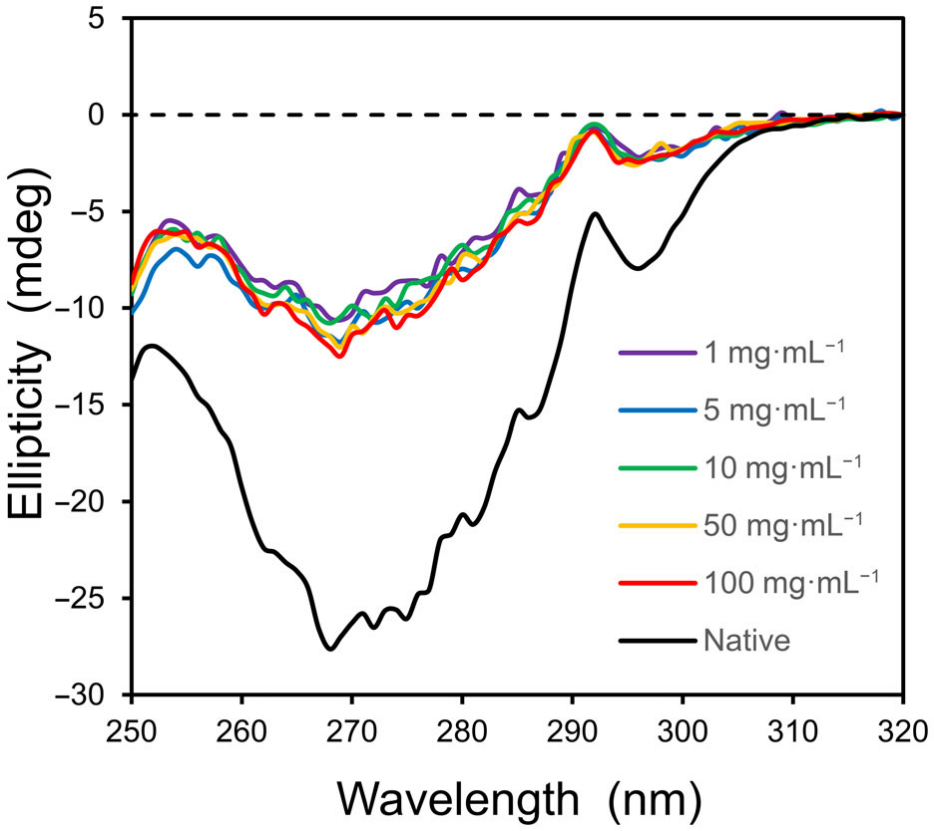
The two‐state equilibrium for α‐lactalbumin in guanidinium chloride does not change with protein concentration. The near‐UV CD profiles of α‐lactalbumin in 1.25 m GuHCl, 10 mm EDTA, and 10 mm Tris at 25 °C are shown as a function of protein concentration. The native structure (lower spectrum) corresponds to 5 mg·mL^−1^ in the absence of GuHCl. The path length of the cuvet (*l*) was varied from 0.01 to 1.0 cm to maintain a constant number of protein molecules in the path of the light source for all samples (*l*·c = 1 cm·mg·mL^−1^).

If desolvation is favorable for the folding transition of α‐lactalbumin (Δ*G* ^S^ < 0), then Eqn (8) predicts that the CD spectra will move toward that of the native folded state with increasing concentration, whereas an unfavorable change in solvation (Δ*G* ^S^ > 0) should trend toward the fully unfolded state, approaching zero ellipticity. As seen in Fig. 6 below, a concentration‐dependent change in tertiary structure is not detected in 1.25 m GuHCl. Upon varying the protein concentration by two orders in magnitude, from 1 to 100 mg·mL^−1^, the corresponding CD spectra are relatively constant. A similar, concentration‐independent set of spectra is observed for α‐lactalbumin in 3 m GuHCl (Fig. S3). These results indicate that the change in solvation energy is approximately zero for this folding equilibrium.

## Discussion

The primary goal of this investigation was to determine the feasibility of applying Eqn (5) to model experimental reactions involving DNA and proteins. The governing equation was applied successfully to the hybridization of two DNA oligonucleotides and to an enzyme‐inhibitor complex, though practical limits in the concentrations of the macromolecules led to large uncertainties in the values of Δ*G* ^S^. For the protein folding model, the observed equilibrium was independent of concentration, suggesting that changes in solvation energy are not a dominant factor. All of these results are discussed below in more detail.

### Solvation in DNA hybridization

The characteristic plots of all hybridization experiments in this study yield a large and positive slope, indicating an unfavorable change in solvation free energy upon duplex formation (Figs 1 and 3B). Although the slopes of the lines in Fig. 3B do not follow a meaningful trend in magnitude with increasing temperature, this is understood to arise from the lack of a correction factor to equate the degree of intramolecular stacking as a function of concentration. For oligo10/10c binding at 25 °C, Δ*G* ^S^ = +460 kcal·mol^−1^ per base pair. Of profound significance to experimental thermodynamics, a modeling study based on the hybridization experiment demonstrates how the classical equilibrium equation can yield a good fit to the raw ITC titration data – but may result in a misleading value for Δ*G*° – when the change in solvation energy is omitted from the governing equation (Fig. 4).

Regarding the magnitude of Δ*G* ^S^ for hybridization, volumetric measurements for a DNA duplex of similar size and composition to oligo10/10c indicate a 25% decrease in solvent‐accessible surface area and the release of ~ 180 water molecules upon duplex formation [19]. Assuming that many of these waters were associated with the ribose–phosphate backbone, their release should lead to an unfavorable change in solvation enthalpy that contributes significantly to the positive sign and magnitude of Δ*G* ^S^. Another possible contributor to the estimated value of Δ*G* ^S^ is the binding or release of counterions along the ribose–phosphate backbone. Salt concentration is known to have a strong influence on the structures of ssDNA and dsDNA [9, 10], and any linked equilibria associated with the movement of counterions will be inherent in the reported value of Δ*G* ^S^ [4].

Of further consideration, hybridization may be a case where the water molecules that remain bound to the reaction product also contribute to the change in solvation free energy. Formation of the double helix is accompanied by a ‘spine’ of hydration in the minor groove, as detected in solution by NMR at 4–10 °C [20], by nonlinear vibrational spectroscopy at room temperature [21], and by all‐atom MD simulations [22]. The degree of hydrogen bonding between water molecules along the spine is known to be weaker for AT‐rich tracts relative to GC‐rich tracts in dsDNA [21], providing one possible reason for the differing values of Δ*G* ^S^ per base pair when comparing oligo9/9c to oligo10/10c. In both cases, the unfavorable change in solvation free energy for duplex formation implicates the displacement of water molecules as a major driving force for the interaction of DNA‐binding drugs and proteins [23, 24].

### Solvation in protein–ligand binding

Understanding the energetic role of water in protein–ligand binding is of fundamental importance to biochemistry and pharmaceutical science [25, 26, 27, 28]. In the current work, the binding of two hydrophilic nucleotide inhibitors with the enzyme RNase A was employed as a model system. The detected changes in equilibria with increasing reactant concentration (Fig. 5) is viewed as an outcome of accounting for the change in solvation energy, in accord with Eqn (5). An unfavorable change in solvation energy for nucleotide/RNase A binding is consistent with (a) a volumetric study that estimates ~ 210 water molecules are released to the bulk upon binding [29] and (b) crystal structures that indicate up to 10 water molecules of low entropy remain hydrogen‐bonded to the inhibitor in the complex [30, 31].

In detailed studies by the Whitesides group, the thermodynamics of ligand binding to carbonic anhydrase was investigated as a model system [32, 33, 34]. The carbonic anhydrase papers focus on hydrophobic interactions and the change in water structure within the enzyme's ligand pocket, yet the authors state that waters in the binding region must be compared to the energetic properties of bulk water, a theme in common with the current work [32]. It is unclear how comparisons of thermodynamic parameters for different ligands obtained from ITC data would be altered – for the Whitesides group and many other experimentalists – if the governing equation used in the current study was employed to obtain Δ*G* ^S^. Should Eqn (5) supplant the classical equation for binding equilibria, then nearly all previous discussions on enthalpy–entropy compensation in protein–ligand binding may become moot; it is difficult to draw a meaningful conclusion regarding the role of water in binding equilibria when the data are interpreted with an equilibrium expression that does not account for the waters released to the bulk phase.

### Solvation in protein folding and the hydrophobic effect

The phenomenon known as the hydrophobic effect has a long and interesting history [35]. It is clear that the hydrophobic effect is an important driving force for protein folding equilibria [36], but the energetic role of water is still a matter of debate. One viewpoint maintains that the hydrophobic effect is driven by the release of high‐energy waters [37, 38, 39], whereas others maintain that high‐energy water cannot exist in a solution at equilibrium [40, 41].

In the current study, the conformation of α‐lactalbumin was observed to be independent of concentration, suggesting Δ*G* ^S^ ≈ 0 for this folding equilibrium. The same result was observed in previous experimental studies for the binding of small nonpolar molecules to β‐cyclodextrin [3, 4]. If hydrophobic interactions, in general, are characterized by negligible changes in solvation free energy, then the α‐lactalbumin result supports the concept that protein folding is driven by an increase in nonpolar–nonpolar interactions.

**Fig. 7:**
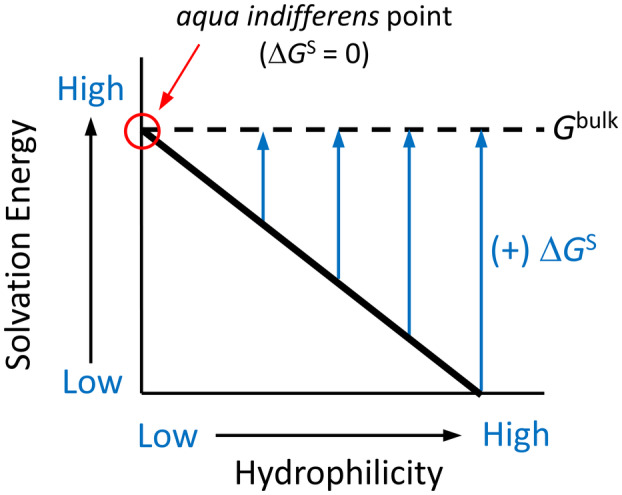
The change in solvation energy for waters released from a surface depends on the average free energy of bulk water. The *y*‐axis represents the solvation energy for a given surface, and the *x*‐axis represents a generic hydrophilicity scale that corresponds to the chemistry of the surface. A nonpolar (low hydrophilicity) surface is characterized by a high solvation energy that approaches the free energy of the bulk water, denoted here as the *aqua indifferens* point (red circle).

Although we concur with the idea that water molecules adjacent to a nonpolar surface are higher in free energy than water near a polar surface, we note that the change in free energy upon release from the surface depends also on the time‐averaged free energy of all (bulk) water molecules in the solution (Eqn 2). The assertion that Δ*G* ^S^ ≈ 0 for hydrophobic interactions does not imply that solvation is unimportant for protein folding and the hydrophobic effect; this view merely places emphasis on the desolvation penalty for polar surfaces, as opposed to a desolvation benefit for hydrophobic surfaces. This concept is conveyed schematically in Fig. 7 by a diagonal line that represents the solvation energy plotted against a generic hydrophilicity scale for the corresponding surface chemistry. Based on our experimental results, as analyzed with a thermodynamic framework that accounts for the contribution of released water molecules, we conclude the free energy of an average bulk water molecule is high (in a dilute solution) and approximately equal to the solvation energy at a hydrophobic surface. In the highly concentrated environment of a living cell, the average free energy of bulk water may be significantly reduced due to the abundance of hydrophilic groups on the surfaces of macromolecules. Thus, the hydrophobic effect may be stronger *in vivo* than observed in a dilute solution.

### Other experimental approaches

Due in part to challenges in monitoring a subset of interfacial water molecules against a background of numerous bulk water molecules, experimental advances in the field of solvation thermodynamics have lagged far behind computational approaches. Water density fluctuations and cavity formation have become computational anthems in deciphering the energetics of hydration [42, 43]. Nonetheless, experimental data related to solvation energies should be highly valued by computational chemists for evaluating their force fields and electrostatic models [44].

One experimental alternative employs molecular torsional balances [45, 46]. Molecular balances are small organic molecules that toggle between two preferred conformations, unfolded and folded. Typically, the conformational equilibrium is monitored by a spectroscopic technique, and the reaction free energy is quantified by the classical approach using Δ*G*° = −*RT*ln*K*. We would encourage synthetic chemists in the field to develop molecular balances of high aqueous solubility, to test the concentration dependence of their conformational equilibria, and to analyze the results with Eqn (8), the same relationship used here for a two‐state folding equilibrium. In addition, it may be interesting to check the concentration dependence of apolar balances in organic solvents; the Piguet group has shown that binding data for the assembly of several lanthanide containers in dichloromethane fit agreeably to Eqn (5) [47, 48, 49, 50, 51].

In a recent set of papers, a technique referred to as terahertz (THz) calorimetry has been described by the Havenith group [52, 53, 54, 55]. In brief, this spectroscopic approach relates difference spectra in the low‐frequency range (Δα), before and after a reaction of interest, to the thermodynamic contribution of two categories of interfacial water, the bound (hydrophilic) subpopulation and the wrap (hydrophobic) subpopulation. Similar to Eqn (2) in the current work, the number of water molecules is unknown for each subpopulation, but their number is proportional to the value of Δα. The energetic contribution of the bound waters is scaled to an experimental enthalpy value, and the contribution of the wrap waters is scaled to an entropy value, allowing one to obtain an estimate for the total change in solvation free energy (Δ*G* ^S^) by summation. When the technique was applied to liquid–liquid phase separation (LLPS) in the presence of the protein elastin, the change is solvation energy was found to vary greatly, depending on the temperature and concentration [55]. For example, at 40 mg·mL^−1^, the solvation free energy changed from a slightly unfavorable value of +11.6 kJ·mol^−1^ at 32 °C to a highly favorable value of −302 kJ·mol^−1^ at 42 °C [55].

The magnitude of the solvation enthalpy and entropy values obtained by THz calorimetry are large compared to typical standard‐state values, but they seem reasonable in comparison to the solvation free energy values obtained by application of Eqn (5) to multiple host–guest binding models [3]. Possible issues for THz calorimetry include questions regarding the calculation of the global free energy change for LLPS, whether a single hydrophilic scaling factor (enthalpy correlation) is appropriate for all polar species including ionic groups of different charge density, and the possibility that buffer salts influence the difference spectra for bound waters in LLPS due to the partitioning of ions between the dilute and condensed phases.

Of significance to the present work, THz calorimetry appears to be amenable to the study of binding equilibria; one could take spectra of the two binding reactants in isolation and then subtract their signals from an equimolar solution of the bound complex, assuming the reaction equilibrium greatly favors product formation. It would be interesting to know if the THz calorimetry approach yields Δ*G* ^S^ values of the same sign and magnitude as any of the previously reported host–guest models [3].

## Conclusions

The experiments in this investigation demonstrate the feasibility of measuring concentration‐dependent changes in equilibria for DNA hybridization and protein–ligand binding. In the case of DNA hybridization, a shift in each tested equilibrium was detectable because the change in solvation energy is large enough in magnitude to overcome the low, sub‐millimolar concentrations of DNA employed. A value for Δ*G* ^S^ of +460 kcal·mol^−1^ per base pair, as obtained for oligo10/10c binding, indicates that the solvation shell of dsDNA is thermodynamically unfavorable relative to the bulk phase and implicates the displacement of water molecules as a major energetic contributor for DNA‐binding proteins and drugs.

The interaction of ribonuclease A with 3′‐UMP was tested as a model for protein–ligand binding and compared to a past result from the literature that used a different nucleotide inhibitor. Binding of both inhibitors led to a positive, unfavorable change in solvation energy when analyzed by Eqn (5). Detecting a change in equilibrium was more challenging for this model system because of the practical limits in maximum protein concentration, coupled with the relatively low value of Δ*G* ^S^ (+160 kcal·mol^−1^ for 3′‐UMP). A conformational study with α‐lactalbumin did not detect a change in equilibrium with increasing concentration, but this result is expected if a near‐zero change in solvation energy is a general characteristic of hydrophobic interactions and if hydrophobic interactions are the dominant force in protein folding equilibria.

This work suggests a fundamental change in the application of thermodynamics to biological reaction equilibria. The scientific community is encouraged to examine other macromolecular interactions as a function of concentration to test further the utility of the governing equations employed here. The technique of THz calorimetry may provide an independent method for evaluating these results. In addition, computational methods that ascertain the chemical potential of water molecules as a function of position [56], both adjacent to and remote from a solute boundary, may supplement experimental approaches and facilitate a deeper appreciation for the role of solvation energy in molecular recognition.

## Conflict of interest

The authors declare no conflict of interest.

### Peer review

The peer review history for this article is available at https://www.webofscience.com/api/gateway/wos/peer‐review/10.1002/2211‐5463.13897.

## Author contributions

CH and DKE designed the experiments for this project. DKE developed the governing equation and wrote the manuscript. All authors contributed to data acquisition and analysis.

## Supporting information


**Fig. S1.** Sample ITC curves for Oligo10/10c duplex formation.
**Fig. S2.** Sample ITC curves for 3′‐UMP/RNaseA binding.
**Fig. S3.** Near‐UV CD profiles of α‐Lactalbumin in 3.00 m GdHCl.
**Table S1.** Thermodynamic values and uncertainties for Oligo9/9c.
**Table S2.** Thermodynamic values and uncertainties for Oligo10/10c.
**Table S3.** Thermodynamic values and uncertainties for 3′‐UMP binding with RNase A.

## Data Availability

ITC data files underlying this study and details of the modeling protocol are openly available by searching the article title in ScholarWorks at https://scholarworks.sjsu.edu/.
